# Non-contrast enhanced functional lung MRI in children: systematic review

**DOI:** 10.3389/fped.2025.1568172

**Published:** 2025-07-22

**Authors:** Carmen Streibel, Grzegorz Bauman, Oliver Bieri, Orso Pusterla, Enno Stranzinger, Marion Curdy, Philipp Latzin, Elisabeth Kieninger

**Affiliations:** ^1^ Department of Paediatrics, Division of Paediatric Respiratory Medicine and Allergology, Inselspital, Bern University Hospital, University of Bern, Bern, Switzerland; ^2^ Graduate School for Health Sciences, University of Bern, Bern, Switzerland; ^3^ Department of General Internal Medicine and Psychosomatic, Heidelberg University Hospital, University of Heidelberg, Heidelberg, Germany; ^4^ Department of Radiology, Division of Radiological Physics, University of Basel Hospital, Basel, Switzerland; ^5^ Department of Biomedical Engineering, University of Basel, Allschwil, Switzerland; ^6^ Department of Diagnostic and Interventional and Pediatric Radiology, Inselspital, Bern University Hospital, Bern, Switzerland

**Keywords:** functional lung MRI, children, lung function, MP-MRI, pulmonology, lung imaging, MRI

## Abstract

**Objectives:**

Magnetic resonance imaging (MRI) of the lung is well suited for repeated measurements especially in children due to the absence of ionizing radiation. Furthermore, non-contrast-enhanced (NCE) functional MRI techniques provide localized functional information on ventilation and perfusion without specialized set-ups (e.g., hyperpolarized gases) using standard clinical MRI systems. Current NCE-MRI techniques in the pediatric setting are matrix-pencil decomposition (MP)-MRI, phase-resolved functional lung (PREFUL)-MRI, self-gated non-contrast-enhanced functional lung (SENCEFUL)-MRI and Fourier decomposition (FD)-MRI. In this article, we comprehensively discuss these innovative techniques.

**Study design:**

We review relevant functional NCE-MRI techniques based on a systematic literature research in MEDLINE, Embase, Cochrane Library, ClinicalTrials.gov and ICTRP. Core concepts were: 1. Aspects regarding lungs 2. MP-, PREFUL-, SENCEFUL and FD-MRI, and 3. children. Consecutively, we included 30 reports.

**Results:**

Functional NCE-MRI in the pediatric setting has been successfully validated and used in observational studies covering a great variety of lung diseases. In contrast to initial implementation studies additionally reporting on clinical findings, later studies focus primarily on clinical topics. Heterogeneous study designs and examination protocols hamper the direct comparability between the different NCE-MRI techniques in terms of their performance against current functional imaging standards or specific objectives.

**Conclusion:**

Their easy applicability makes NCE-MRI techniques highly attractive for widespread clinical use. Following successful implementation studies, still varying test protocols and approaches for calculating outcome values must next be compared and standardized.

## 1 Introduction

High-quality imaging of the lung using magnetic resonance imaging (MRI) is challenging as the lung is an air-filled organ in constant motion, but recent technical improvements enabled successful implementation (1–5). Since patients are not exposed to any ionizing radiation, MRI of the lung is well-suited in the pediatric setting and for repeated follow-up measurements of chronic lung diseases (6–8). In comparison, computer tomography (CT) examinations of the lungs are shorter. However, the young patient is still exposed to 0.06–0.15 mSv in ultra-low-dose CT with photo-counting (9, 10) up to 0.5–1.5 mSv in low-dose CT (11), requiring a benefit-risk assessment and/or justification, especially in young patients (12). While CT remains superior for detailed morphological assessment, especially for bronchiectasis and ground glass opacities, MRI performs well in visualizing bronchial wall thickening, mucus plugging, and air trapping (5, 13–18). An innovative possibility to combine the benefits of MRI (no radiation) with the high spatial resolution and low noise characteristics of CT is the use of synthetic images (19). In this approach, deep-learning techniques are exploited to generate CT-like images from MRI-data (19). In our study, we did not use these techniques, but focused on functional imaging. In our study, we did not use these techniques, but focused on functional imaging.

Besides structural scans providing morphological information, specifically tailored functional MRI techniques allow to assess spatially resolved pulmonary function, such as local ventilation and perfusion (1, 2, 20). Notably, non-contrast-enhanced (NCE) functional MRI techniques (21–31) do not require specialized set-ups, unlike lung MRI with hyperpolarized gases (e.g., ^129^Xenon or ^3^Helium MRI) (2) or dynamic-contrast-enhanced (DCE) MRI (1) (Table 1). Most importantly, as the scans are performed during free tidal breathing, there is no need for breathing maneuvers or breath-holds. This aspect is extremely important especially in young children and leads to much higher success rates of these techniques at that crucial age. This ease of application makes NCE-MRI highly attractive for broad clinical use, also in contrast to CT scans, which do not allow assessment of lung perfusion without intravenous contrast agents. Additionally, ultrashort echo time (UTE) or StarVibe sequences enable to visualize structural lung aspects including atelectasis and fibrosis. However, this upcoming technique is not necessarily well known to pediatricians outside the highly-specialized field. Thus, our article provides an attractive mix of a clear introduction to the technical background, as well as a systematic literature review on the relevant functional NCE-MRI techniques for lung imaging in the pediatric field.

**Table 1:** Overview over different types of lung MRI.

	Structural MRI	Hyperpolarized Gas MRI	Dynamic contrast enhanced MRI	Non-contrast-enhanced MRI
Assessing	Morphology	Ventilation, gas diffusion	Perfusion	Ventilation and perfusion
Requirement	None, in special cases gadolinium based i.v. contrast agent if needed	Hyperpolarized gas for inhalation	Gadolinium-based i.v. contrast agent	None
Relevant Subtypes	Different imaging techniques to assess the lung structure including 2D/3D T1-, T2-weighted, fat-saturated or contrast-enhanced pulse sequences	^129^Xenon MRI	T1-weighted 3D + t spoiled gradient echo acquisitions	MP
		^3^Helium MRI		PREFUL
				SENCEFUL
				FD
Approx. duration of Examination	10–20 min^a^	15–30 min^b^	1–15 min^c^^,^^d^	7.9 + - 1.8 min (MP)^a^
Advantages	- Easily applicable on clinical MRI-scanners - Well-established	- Additional measure of gas diffusion also possible - Additional measure of gas exchange efficiency using the dissolved-phase of ^129^Xenon in the lung also possible	- Well-validated - Additional quantification of hemodynamic parameters possible	- Easily applicable on clinical MRI-scanners - Non-invasive - Assessment of regional ventilation and perfusion in parallel - Repeated measures also feasible for patients at risk (children with chronic disease, etc.)
Disadvantages	- The local function can only be inferred from the morphological state of the lung	- Expensive, highly specialized set-up needed - Patients need to be able to perform specific breathing maneuvers - No direct assessment of perfusion maps	- Invasive, contrast agent needed - Some patient groups excluded (e.g., highly reduced kidney function) - Only assessment of perfusion	- Limits of normal for outcome values not available for all techniques - Partially still changing examination protocols/work in progress - Except for k-means classification, local impairment of lung function is not determined in absolute terms, but relative to the patient's remaining depicted lung area (not a direct measurement of ventilation or perfusion)

MRI, magnetic resonance imaging; i.v, intravenous; 2D, two-dimensional; 3D, three-dimensional; 3D + t, three-dimensional + time; MP-MRI, matrix-pencil decomposition MRI; PREFUL-MRI, phase-resolved functional lung MRI; SENCEFUL-MRI, self-gated non-contrast-enhanced functional lung MRI; FD-MRI, fourier decomposition MRI.

^a^ Willers CC, BS Frauchiger, E Stranzinger et al. (2022) Feasibility of unsedated lung MRI in young children with cystic fibrosis. Eur Respir J DOI: 10.1183/13993003.03112-2021.

^b^ Stewart NJ, LJ Smith, HF Chan et al. (2022) Lung MRI with hyperpolarised gases: current & future clinical perspectives. Br J Radiol DOI: 10.1259/bjr.20210207.

^c^ Zöllner FG, K Zahn, T Schaible, SO Schoenberg, LR Schad, KW Neff (2012) Quantitative pulmonary perfusion imaging at 3.0 T of 2-year-old children after congenital diaphragmatic hernia repair: initial results. Eur Radiol DOI: 10.1007/s00330-012-2528-9.

^d^ Groß V, K Zahn, K Maurer et al. (2022) MR lung perfusion measurements in adolescents after congenital diaphragmatic hernia: correlation with spirometric lung function tests. Eur Radiol DOI: 10.1007/s00330-021-08315-9.

### 1.1 General information on non-contrast enhanced functional lung MRI

We focus on the most relevant NCE-MRI techniques implemented in the pediatric setting: Fourier decomposition (FD)-MRI (21), matrix-pencil decomposition (MP)-MRI (22), phase-resolved functional lung (PREFUL)-MRI (24) and self-gated non-contrast-enhanced functional lung (SENCEFUL)-MRI (30).

**Figure 1:**
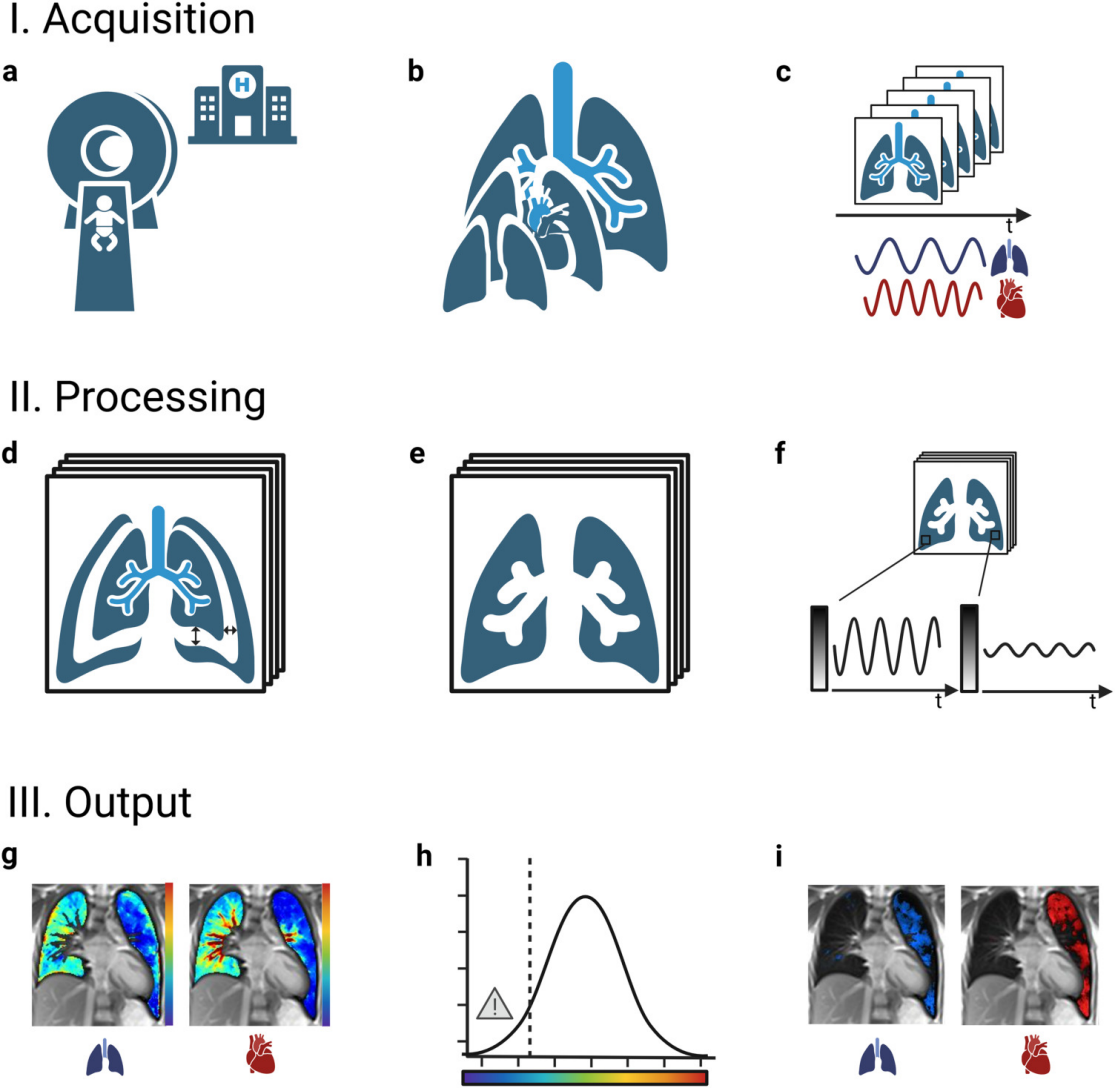
General workflow of functional non-contrast-enhanced MRI scans of the lung. I.: A standard clinical MRI scanner without any specialized set-up is used **(a)** to examine the lung at multiple slice locations **(b)**. At each slice location, a time resolved image series is acquired during ongoing breathing and heartbeat **(c)**. II.: The images are of each series are adjusted for respiratory motion (registration) **(d)** and the image area depicting lung tissue is selected (segmentation) **(e)**. Periodic signal intensity modulations over time caused by respiration and blood-flow are analyzed voxel wise **(f)**. III.: As the amplitudes of the modulations correspond to regional ventilation and perfusion, quantitative color maps can be calculated **(g)**. Areas with a level of ventilation or perfusion below a certain threshold are marked as impaired (classification) **(h)**, returning defect maps and the affected relative lung volume (VDP, ventilation defect percentage; QDP, perfusion defect percentage) as numeric outcome values **(i)**. MRI, magnetic resonance imaging.

For all these techniques, measurements are performed on standard clinical MRI scanners [usual field strength 1.5 Tesla (T) or 3 T, also promising preliminary results at low field of 0.55 T (32–35)] without the need of contrast agent, inhalation of hyperpolarized gases or specific breathing maneuvers. The usual acquisition time of a comprehensive lung MRI protocol including morphological and functional sequence is about 20–30 min. To ensure short examinations, limiting morphological sequences to the most relevant ones is convenient. As an example, at our centre, the application of T2 weighted HASTE (Half Fourier Acquisition Single Shot Turbo Spin Echo) sequences in transversal and coronal direction with 5 mm slice thickness each and of T2 weighted UTE 3D Spiral VIBE with 1.25 mm isotropic voxel size in coronal direction takes approx. 10 min. Regarding functional NCE-MRI techniques, the examination time for all techniques has so far mainly been dependent on the number of slices acquired (acquisition time per slice approx. 1–3 min). Accordingly, MP-MRI examinations covering of the whole lung (12 slices) take approx. 19 min (36), PREFUL examinations including 7 slices require 5–11 min (varying temporal resolution) (37) and SENCEFUL examinations of 6 slices take about 16 min (38). Shorter acquisition times are achievable by reducing the number of slices assessed [e.g., 1:16 min for one central slice in PREFUL (39)] or by applying UTE sequences: 3D UTE-SENCEFUL sequences take 8.7 min to cover the whole lung with current research pushing the acquisition time to 5 min and shorter (38). The scans are performed with a high success rate in unsedated children from the age of 5 years onwards (36) and are feasible in newborns during natural sleep (39, 40). In older children, hearing protection is provided by earplugs and earmuffs. In newborns, a “feeding and swaddling technique” is used, including prior feeding, swaddling with blankets and hearing protection with earplugs, earmuffs and blanket padding (39, 40). Depending on whether a particular central section or the entire lung volume is examined, single-slice, multi-slice or three-dimensional (3D) approaches can be applied. At each requested slice location, a time-resolved image series is acquired during free tidal breathing. This allows recording respiratory and cardiac signal modulations over time (Figure 1: I. Acquisition). The acquired image series are further processed (Figure 1: II. Processing) and compensated for respiratory motion (non-rigid image registration) (41, 42). The areas depicting lung tissue are selected either manually or automatically (segmentation) (43, 44) for further analysis. Within an image series over time, MRI signal changes are caused by respiration and perfusion: as for respiration, the expansion of lung alveoli during inspiration leads to a decrease in T2 tissue signal intensity due to the lack of 1H in the inhaled air (very low echo time of <1 ms required to ensure susceptibility). Regarding perfusion, the influx of unsaturated blood causes a decrease in T2 signal intensity due to dephasing effects of paramagnetic deoxyhemoglobin on nearby H_2_O molecules. These observed modulations of signal intensity within the image series are analyzed voxel-wise in the frequency range of respiration (expansion of the lungs with decrease in tissue intensity) and pulsation (influx of unsaturated blood). Their amplitudes reflect the regional level of ventilation and perfusion, which allows calculating quantitative color-coded maps of the lung (Figure 1: III. Output).

To derive quantitative outcome measures from the information displayed on the functional color-coded maps, the most common approach is semi-quantitative (1). It determines the percentage of lung volume that is considered functionally impaired (classification). Lung areas classified respectively show decreased ventilation or perfusion compared to the remaining (normal/healthy) lung or relative to an external healthy reference group [ventilation defect percentage (VDP), perfusion defect percentage (QDP), percentage of combined/matched ventilation and perfusion defect (VQM, also called VQD_match_)] (1). The affected areas are visualized on corresponding defect maps. Different computational methods are applied to calculate the respective threshold below which the regional level of ventilation or perfusion is classified as impaired, e.g., k-means (45), linear binning (46) or fractional mean or median signal (47, 48). Therefore, a direct comparison of VDP and QDP values from different MRI techniques and data is only possible to a limited extent. Further, study protocols vary with regard the number of slices acquired [from a single central slice (33, 39, 40, 49–51) to multiple slices (30, 37, 40, 52–55) even covering the entire lung (44, 47, 48, 52, 56–66) or 3D approaches (39, 67, 68)]. An additional outcome parameter, the defect distribution index (DDI), allows assessing the scattering or clustering of the impaired lung regions (56). This means, that one large defect results in a higher DDI compared to several small defects of the same overall extent (56).

### 1.2 Details on technical background of non-contrast enhanced functional lung MRI

All functional NCE-MRI techniques exploit the principles elaborated above and do in general employ steady-state free precession pulse (SSFP) sequences for data acquisition including balanced SSFP (bSSFP) and spoiled gradient recalled echo (SPGR) pulse sequences (22, 24, 30, 34, 35). In general, the functional NCE-MRI techniques can work with either SPGR and bSSFP data. However, due to the different calculation methods behind each technique, some sequences and field strengths are particularly advantageous and established.

- FD-MRI is one of the first approaches of functional NCE-MRI, from which all other techniques above are derived (1, 21). For scans, a bSSFP pulse sequence is usually used. To ensure high signal intensity of the pulmonary parenchyma, a short repetition time is crucial. This can be achieved by a high bandwidth of 1,447 Hz/pixel and an asymmetric echo sampling. In addition, the image sampling frequency needs to be sufficient for the subsequent spectral analysis and an acceptable image resolution is needed. Empirically, an acquisition rate of 3.33 images/s was shown to be a good compromise in this regard. As next steps, image registration (44) is followed by voxel-wise Fourier decomposition of the dynamic lung image series as method of spectral analysis of the signal modulations (21). The amplitudes derived from the main spectral lines represent the signal from lung parenchyma and pulsatile pulmonary blood. The spectral lines are susceptible to irregularities in the respective respiratory and pulsatile rates: As perfusion and ventilation spectral lines are widened, a larger frequency range hat to be integrated and thus more noise is present in the calculated images.
- MP-MRI is especially robust against artifacts due to both, the applied pulse sequence in acquisition and the exploited signal decomposition method. First, advantageously high signal-to-noise ratio (SNR)—outstanding also compared to all other NCE-MRI techniques—in the lung tissue is ensured by mainly utilizing an ultra-fast balanced steady-state free precession (uf-bSSFP) pulse sequence for data acquisition (22, 34, 35). The shortened repetition time applied in this sequence leads to improved signal intensities, decreased image acquisition time and decreased motion and banding artifacts. However, uf-bSSFP is especially well-suited for acquisitions at 0.55 T or 1.5 T, but its use at 3 T is limited. Here, a transient SPGR is a better choice for MP-MRI at 3 T (34). Post-processing includes non-rigid image registration (41) and automated segmentation of the lung with recognition of the individual lung lobes (43, 45, 46). Maps of respiration, perfusion, and blood arrival time are generated from the acquired respiratory and cardiac signal modulations applying voxel-wise matrix pencil decomposition as method of spectral analysis (21, 22, 35). The lung signal intensity is summed up in the automatically segmented lung tissue and then low and high-pass filtered to separate respiratory and cardiac cycles. As a result, MP-MRI, as compared to FD-MRI, is more robust against truncation artefacts. Further, the length of the time-resolved image series has less impact on the estimated amplitude values.
- PREFUL-MRI usually exploits a spoiled gradient recalled echo (SPGR) pulse sequence for data acquisition at 1.5 T or 3 T (24). Additionally, for examinations on 1.5 T or 0.55 T low-filed scanners a bSSFP sequence can be used, as for FD-MRI and MP-MRI (69). After image registration, the separation of cardiac and respiratory signal modulations is achieved by applying FD analysis. In the subsequent post processing, a low-pass filter is used and afterwards the images of the time-resolved series are assigned to a single cardiac or respiratory cycle according to their estimated phase. When calculating cardiac phase positions, the time series is subdivided based on inherent local maxima and a piecewise sinusoidal fit to avoid artifacts caused by irregularities in the pulsatile frequency. Regarding the respiratory cycle, the slope of the plotted respiratory signal intensities over time is used to divide the images into inspiratory and expiratory phase and assign their phase positions. Subsequent reconstruction of complete cycles [based on Nadaraya-Watson kernel (nonparametric) regression using a Gaussian kernel (sigma = 0.1)] to determine the modulation amplitudes allows ventilation and perfusion maps to be computed based on higher temporal resolution than the original sampling rate of the data (24). Disadvantageously, lacking quasi-random acquisition, repeated collection of the same phases may occur in PREFUL-MRI scans in case of acquisition/ cardiac synchronization.
- SENCEFUL-MRI utilizes mainly a SPGR pulse sequence with quasi-random phase encoding combined with an acquisition of non-phase-encoded signals for respiratory and cardiac self-gating (30). After low-pass filtering, the acquired non-phase-encoded gating signal over time is applied to separate ventilation and perfusion signal modulations using Fourier decomposition. Next, the absolute position within the respiratory or cardiac cycle is determined for each image (and each image date point accordingly) of the time series: Regarding the respiratory cycle, the gating signal data of a coil element near the diaphragm affected by the diaphragmatic movement and thus reflecting signal modulations related to respiration is further analyzed. Considering the slope of the gating signal series, inspiration and expiration can be distinguished and respiratory cycles, as found between two equally oriented turning points, can be obtained. Regarding perfusion, gating signal variations over time of a coil element close to the cardiac arch and thus affected of pulsatile blood flow are analyzed to obtain cardiac cycles. Subsequently, the quasi-randomly acquired data can be binned to individual cardiac/respiratory phases by shifting gating windows over the identified cycles. This allows the reconstruction of complete representative cardiac and respiratory cycles per voxel and the computation of color-coded maps based on the according signal change amplitudes (30). One limitation to note is that the images can only be reconstructed in diastole.

## 2 Review based on systematic literature research

### 2.1 Search methods and selection criteria

**Figure 2:**
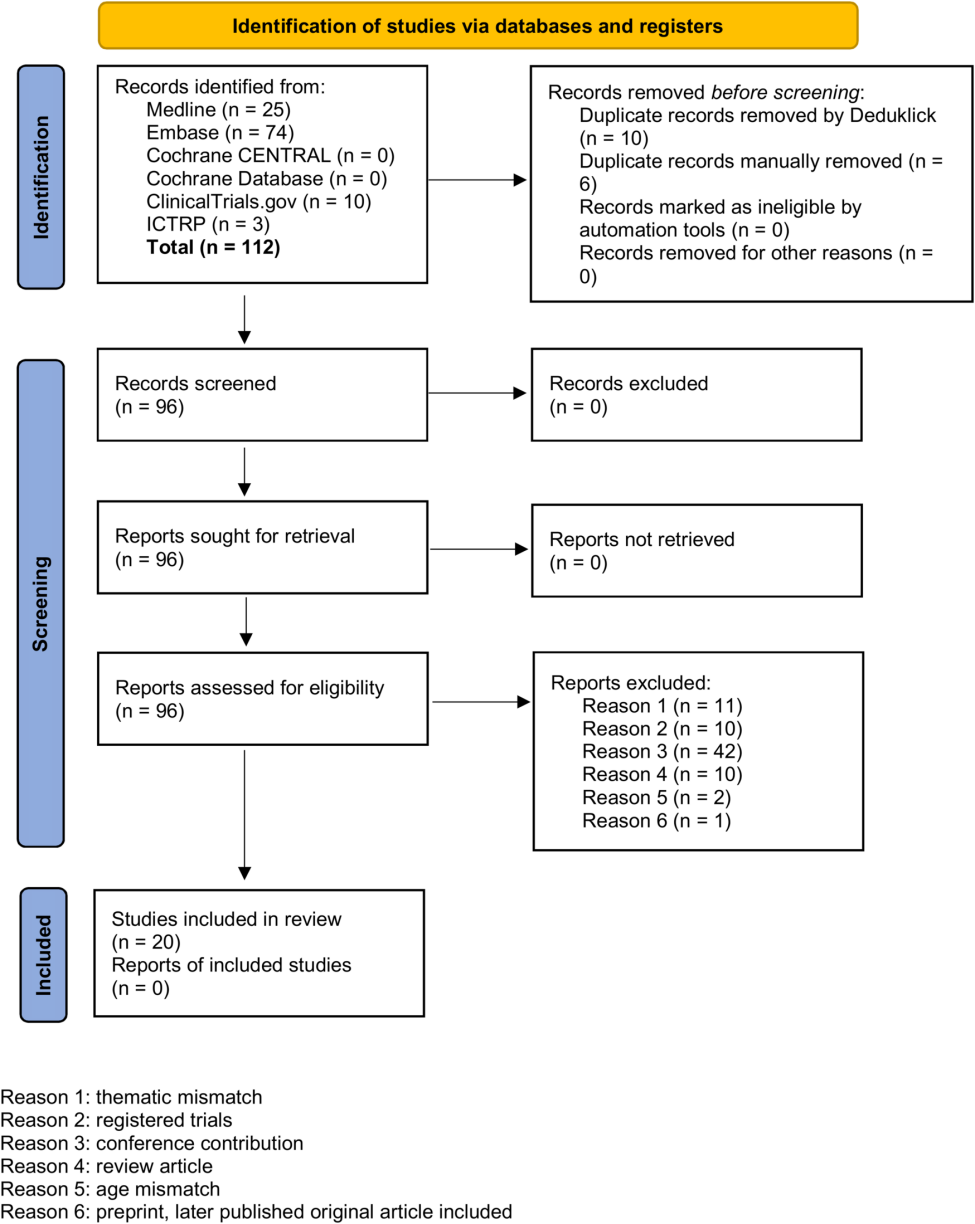
PRISMA 2020 flow diagram.

We used the following information sources for our literature research without applying any language restrictions: MEDLINE® ALL (Ovid), Embase (Ovid), Cochrane Library (Wiley), ClnicalTrials.gov, International Clinical Trials Registry Platform ICTRP. For all databases, the data of last request was 12th of August 2024. We aimed to find publications on MP-MRI, PREFUL-MRI, SENCEFUL-MRI or FD-MRI for functional imaging of the lung in children and used appropriate search strategies. Of 96 reports assessed for eligibility, we excluded 76 reports due to duplicates, thematic or age mismatch or because the findings were records on registered trials, conference contributions or review articles highlighting the functional MRI techniques of interest only in small subsections. In order not to overlook any publications in this innovative research area with evolving names and key phrases, all included articles were also screened for relevant references cited to add to the set (*n* = 10). Figure 2 shows a PRISMA diagram of the search and inclusion strategy. Further details on the search strategy are presented in the online Supplementary Material (OLS). An overview over the 30 included reports is provided in Table 2 and OLS Table 1.

**Table 2:** Overview over published reports on functional NCE-MRI of the lung applied in the pediatric setting.

Disease group/NCE-MRI type	MP-MRI	PREFUL-MRI	SENCEFUL-MRI	FD-MRI
Cystic Fibrosis	• Validation of Fourier decomposition MRI with dynamic contrast-enhanced MRI using visual and automated scoring of pulmonary perfusion in young cystic fibrosis patients (47) • Novel magnetic resonance technique for functional imaging of cystic fibrosis lung disease (48) • Ventilation and perfusion assessed by functional MRI in children with CF: reproducibility in comparison to lung function. (65) • The impact of segmentation on whole-lung functional MRI quantification: Repeatability and reproducibility from multiple human observers and an artificial neural network (44) • Defect distribution index: A novel metric for functional lung MRI in cystic fibrosis (56) • Effect of Salbutamol on Lung Ventilation in Children with Cystic Fibrosis: Comprehensive Assessment Using Spirometry, Multiple-Breath Washout, and Functional Lung Magnetic Resonance Imaging (57) • Effects of elexacaftor/tezacaftor/ivacaftor therapy in children with cystic fibrosis—a comprehensive assessment using lung clearance index, spirometry, and functional and structural lung MRI (64) • Contrast agent-free functional magnetic resonance imaging with matrix pencil decomposition to quantify abnormalities in lung perfusion and ventilation in patients with cystic fibrosis (63)	• Functional lung MRI for regional monitoring of patients with cystic fibrosis (54) • Feasibility of quantitative regional ventilation and perfusion mapping with phase-resolved functional lung (PREFUL) MRI in healthy volunteers and COPD, CTEPH, and CF patients (24) • Flow Volume Loop and Regional Ventilation Assessment Using Phase-Resolved Functional Lung (PREFUL) MRI: Comparison With (129) Xenon Ventilation MRI and Lung Function Testing (53) • Comparison of Functional Free-Breathing Pulmonary (1)H and Hyperpolarized (129)Xe Magnetic Resonance Imaging in Pediatric Cystic Fibrosis (49) • Free-breathing MRI for monitoring ventilation changes following antibiotic treatment of pulmonary exacerbations in paediatric cystic fibrosis (51) • A dual center and dual vendor comparison study of automated perfusion-weighted phase-resolved functional lung magnetic resonance imaging with dynamic contrast-enhanced magnetic resonance imaging in patients with cystic fibrosis (52) • Xe and Free-Breathing H Ventilation MRI in Patients With Cystic Fibrosis: A Dual-Center Study. Journal of Magnetic Resonance Imaging (37) • Inter- and intravisit repeatability of free-breathing MRI in pediatric cystic fibrosis lung disease (50) • Effect of CFTR modulator therapy with elexacaftor/tezacaftor/ivacaftor on pulmonary ventilation derived by 3D phase-resolved functional lung MRI in cystic fibrosis patients (67) • PREFUL MRI for Monitoring Perfusion and Ventilation Changes after Elexacaftor-Tezacaftor-Ivacaftor Therapy for Cystic Fibrosis: A Feasibility Study (70)	• Self-gated Non-Contrast-enhanced Functional Lung MR Imaging for Quantitative Ventilation Assessment in Patients with Cystic Fibrosis (71) • Non-contrast pulmonary perfusion MRI in patients with cystic fibrosis (58)	• Non-Contrast-Enhanced Functional Lung MRI to Evaluate Treatment Response of Allergic Bronchopulmonary Aspergillosis in Patients With Cystic Fibrosis: A Pilot Study (55)
Bronchopulmonary dysplasia and Fetal Growth Restriction	• Low Birth Weight and Impaired Later Lung Function: Results from a Monochorionic Twin Study (62)	• Assessment of lung ventilation of premature infants with bronchopulmonary dysplasia at 1.5 Tesla using phase-resolved functional lung magnetic resonance imaging (40)		• Lung structure and function on MRI in preterm born school children with and without BPD: A feasibility study (59)
Other pulmonary diseases	• Structural and Functional Lung Impairment in Primary Ciliary Dyskinesia. Assessment with Magnetic Resonance Imaging and Multiple Breath Washout in Comparison to Spirometry (60)	• Clinical Feasibility of Structural and Functional MRI in Free-Breathing Neonates and Infants (39) • Pulmonary Dysfunction after Pediatric COVID-19 (33)		
Localized lung alterations	• School-age structural and functional MRI and lung function in children following lung resection for congenital lung malformation in infancy (61) • Long-term pulmonary outcome of children with congenital diaphragmatic hernia: functional lung MRI using matrix-pencil decomposition enables side-specific assessment of lung function (66)		• SElf-gated Non-Contrast-Enhanced FUnctional Lung imaging (SENCEFUL) using a quasi-random fast low-angle shot (FLASH) sequence and proton MRI (30)	

### 2.2 Summary of findings

In most publications, the initial research question was defined broadly and a large number of findings were presented in parallel. In contrast to the initial implementation studies, which additionally report on clinical findings, later studies focus primarily on clinical topics and secondarily cover technical topics.

#### 2.2.1 Implementation studies and results on technical aspects of further studies

Initial studies demonstrated the feasibility of MP-, PREFUL-, SENCFUL- and FD-MRI to assess regional lung function, mainly in patients with cystic fibrosis (CF) compared to healthy controls (24, 47, 49, 53, 54, 58, 65, 71). High overall concordance with hyperpolarized gas MRI (^129^Xe) (37, 49–51, 53) and DCE-MRI (24, 30, 47, 52, 54, 63) was shown regarding both, the spatial overlap between the lung regions classified as impaired and the concordance of derived numerical outcome values (e.g., elevated DP in patients with CF). Classification methods and specific methods used to quantify agreement of outcomes differed due to heterogeneous study designs: To be more detailed, the OLS Supplementary Table S1, column “classification”, lists the respective computational methods used to calculate respective thresholds below which the regional level of ventilation or perfusion is classified as impaired, such as k-means, linear binning or fractional mean or median signal multiplied by empirically defined factors. Various mathematical and statistical approaches were applied to validate the agreement between NCE-MRI and hyperpolarized gas MRI or DCE-MRI examinations and the respective defect maps or outcome values (e.g., correlation of DPs, dice coefficients, differences between functional scores). Further criteria used to compare NCE-MRI with the already established techniques of functional lung imaging, were the congruence of the diagnostic information provided (e.g., the discrimination between healthy and sick individuals) or the visual agreement of the initial color maps themselves. Thus, this heterogeneity hampers the comparability between the different NCE-MRI techniques in terms of their performance against current functional imaging standards. The same applies to the reported association between the NCE-MRI outcome values and frequently used lung function outcomes (37, 44, 48–51, 54–56, 58, 60, 62, 63, 65–67, 70, 71), reproducibility (44, 50, 52, 65) and response to treatment (51, 55, 57, 64, 67, 70). Regarding pulmonary function tests, the main outcome parameters compared with NCE-MRI are “forced expiratory volume in one second” (FEV_1_, converted into percentage predicted value or z-score) and “lung clearance index” (LCI). Most studies show moderate to strong correlations (VDP_FEV_1_ −0.51 to −0.85, VDP_LCI 0.5–0.82, QDP_FEV_1_ −0.67 to −0.78, QDP_LCI 0.77–0.85) (37, 44, 48–51, 55, 56, 58, 63, 65–67, 70, 71). Weak or missing significant correlations are rarely reported (50, 54, 60, 62). However, the topic is mainly only investigated as an additional endpoint. In studies primarily focusing on reproducibility, a good (0.87) to excellent (0.97) intraclass correlation coefficient is reached for repeated measures within 24 h (50, 65).

In the further course, numerous refinements and additional functions have been continuously developed and implemented, such as 3D scans (39, 67) and sequences already feasible in neonates (39, 40). The introduction of automatic segmentation of the area depicting the lung tissue on the MRI images has considerably simplified further data processing (44). For MP-MRI, e.g., an automated processing pipeline (TrueLung) is available as a docker container running a Linux operating system. The total calculation time required per subject is 20 min (72). With regard to the derivation of outcome values, qualitative evaluation scales (47, 55, 58) as well as various classification approaches (24, 30, 33, 37, 39, 40, 44, 47–63, 65–67, 70, 71) and additional quantitative measures (flow-volume loop, mean fractional w quantitative measures (flow-volume loop, mean fractional ventilation) (24, 30)were investigated. Today, defect percentages (DP) are the main outcome parameters used, although a consensus on one classification approach, i.e., exact computational method for calculating the respective thresholds, is pending.

Within the findings of our literature search, there were no explicit superiority/non-inferiority/equivalence trials comparing one NCE-MRI technique to another NCE-MRI technique or further (functional) imaging techniques with regards to clinimetric properties (73, 74) or patient relevant outcomes. This also applies to the options for the number of slices acquired, the parameters assessed and the classification methods used, even within each single NCE-MRI technique.

#### 2.2.2 Clinical observational studies and results on clinical aspects of further studies, sorted by disease group

##### 2.2.2.1 Cystic fibrosis

In studies conducted prior to the introduction of cystic fibrosis transmembrane conductance regulator (CFTR) modulator therapy, children with CF showed an increased ventilation and perfusion impairment compared to healthy controls (24, 47–49, 53, 54, 58, 65, 71), especially those having frequent pulmonary exacerbations (37, 49, 51). When monitoring treatment responses, a decrease of ventilation and perfusion defects was observed after intravenous antibiotic therapy of acute pulmonary exacerbations or after treatment of allergic bronchopulmonary aspergillosis (51, 55). Further studies reported on improvements after inhaled hypersaline therapy (54) or salbutamol therapy (57). A long-term decrease of lung ventilation and perfusion defects with resolving of cluster impaired areas upon triple-CFTR-modulator therapy was shown recently (64, 67, 70).

##### 2.2.2.2 Bronchopulmonary dysplasia and fetal growth restriction

NCE-MRI performed in neonates with bronchopulmonary dysplasia showed increased VDP (40), and examinations at school-age revealed sporadic persistent ventilation and perfusion impairments (59). In 6- to 18-year-old children with fetal growth restriction, no abnormalities were found using functional MRI imaging despite reduced spirometric lung volumes (62).

##### 2.2.2.3 Other pulmonary diseases

NCE-MRI was additionally used to investigate other pulmonary diseases such as primary ciliary dyskinesia (increased VDP and QDP) (60) and after SARS-CoV-2 infection (increased VDP) (33).

##### 2.2.2.4 Localized lung alterations

The fact that NCE-MRI can identify lung regions with impaired ventilation or perfusion seems promising in children with suspected localized alterations. Results e.g., showed local functional impairment (i) in the expanded lung tissue of children following lung resection for congenital lung malformation in infancy (61), (ii) in the affected lung of patients with a large congenital diaphragmatic hernia (66) or (iii) in an adolescent with hypoplasia of the left pulmonary artery (30).

The clinical studies were all observational studies or studies comparing patients to healthy controls (24, 30, 33, 39, 40, 47–49, 53, 54, 58, 59, 62, 65, 71). NCE-MRI has not yet been used as primary outcome parameter in randomized controlled trials, but several studies exist that have reported. changes in NCE-MRI outcomes upon different interventions in children (51, 55, 57, 64, 67, 70).

## 3 Discussion

Beyond valuable pioneer work, which demonstrated the feasibility of functional NCE-MRI of the lung, an increasing number of studies exist proving its applicability in children with various diseases and within a wide age range.

Primary validation and good comparability to the gold standards of functional imaging of the lung was successfully done for all functional NCE-MRI techniques presented (24, 30, 37, 47, 49–54, 63). It is important to point out that the validation of the individual techniques was in part also carried out in studies including only adults or in animal experiments (21, 24, 30, 75). Additionally, studies in adults cover further diseases including asthma and COPD (24, 76). Due to our search strategy, these studies are not listed in Table 2. In addition, the enormous development potential of NCE-MRI with regard to a wide range of optional applications has already become clear in various projects, for example through adaptation for use in newborns (39, 40), on devices of different manufacturers (37, 52) or low-field devices (33, 77). However, for some NCE-MRI techniques, an assessment of the feasibility of these approaches is still pending. Further, possible future technical developments taking into account the innovative real-time MRI (78) might be promising.

The most important future problem to solve seems to provide guidance on the variety of acquisition protocols and outcome calculation models (i.e., calculation basis of VDP and QDP, variations also presented in OLS Supplementary Table S1, column classification) both within and between the different NCE-MRI techniques. Heterogeneous study designs and different local set-ups prevent direct comparability of numeric endpoints. This seems especially disadvantageous as in childhood sample sizes are rather small and only few intervention studies assessing treatment effects with NCE-MRI techniques exist. The ultimate aim should be to reach an expert consensus within the research community on which benchmarks need to be met when using NCE-MRI to ensure conformance to current reference standards. Ideally this would be done—as for other techniques—in workgroup meetings and consensus guidelines endorsed by the respective societies. Another approach might be to conduct sufficiently powered superiority studies on aspects of technical implementation that assess clinimetric properties (73, 74) as endpoints of the various approaches. For this purpose, re-analyzing previously performed measurements of the respective research groups might be considered to exploit the data already collected. Subsequently, this should allow to develop one single acquisition protocol and analysis approach or even to identify a preferred NCE-MRI technique. Specifically, various aspects requiring further investigation can be identified within the areas of data acquisition and output calculation. With regard to data acquisition, the open questions remaining concern the number of slices (one central slice vs. up to multiple slices covering the whole lung) most advantageous as well as the most suitable use of specific field strength and sequences. So far, applicability on low-field devices seems advantageous due to the lower costs and the better portability of the corresponding devices and hence the easier imaging accessibility (79). As for output calculation, varying approaches exist regarding when lung regions are considered/ classified as functionally impaired and regarding the conversion of defect maps into numerical outcome values (a.o. QDP, VDP, DDI, flow-volume loop). Despite the lack of sufficiently powered superiority studies on classification methods to date, the linear binning approach (80) appears to be particularly promising from a methodological point of view. In contrast to other threshold-based classification methods, the linear binning method assigns a lung area as “impaired” in terms of ventilation or perfusion not in comparison to the remaining, non-affected lung tissue depicted on the respective MRI slice, but relative to an external healthy reference group. Especially in diseases with a homogeneous decrease in total lung function, e.g., BPD, the linear binning classification approach might be important to avoid false negative results caused by the lack of localized impairment. In addition, the individual bins/outcome categories in the linear binning approach (defected, lowered, normal and elevated ventilation or perfusion) enable a more differentiated sub-analysis than a pure distinction between defect (corresponding proportion quantified in defect-percentage) vs. non-defect lung regions. However, it should be noted that the reference values should be based on data from a control group of large sample size to better compensate for natural variability. In this regard, the already existing collection of a correspondingly large data set, as known for MP-MRI with more than 900 measurements (81), appears particularly promising. As a further aspect, Another aspect is that the percentage of combined/matched ventilation and perfusion defects (VQD_match_) could be a particularly robust numerical outcome value due to the parallel consideration of ventilation and perfusion impairment. Broader application of NCE-MRI, allowing for multi-site studies with large sample sizes and devices from different manufacturers, will be crucial to fully exploit the innovative potential already apparent in the studies conducted so far.

Regarding the benefits of using functional NCE-MRI of the lung, various case studies illustrate its potential for clinical decision-making (81). The potential of obtaining visual and numerical information on ventilation and perfusion of the lung without contrast and during tidal breathing seems enormous, and also with this article we aim to raise awareness among clinicians. Besides others, the advantages of the technique include: identifying and visualizing exact lung areas affected by underlying disease and/or to specifically examining the extent and level of functional impairment. Indications for therapeutic interventions can be determined accordingly (e.g., bronchoscopic removal of mucus plugging). Further, NCE-MRI allows a better understanding of lung pathophysiology in complex diseases by assessing and visualizing local lung function, in contrast to global lung function tests measured at the mouth. Thus, among others, patients with side-related anatomic aberrations or poorly-understood respiratory symptoms, such as in sickle cell disease, can profit directly from NCE-MRI examinations. At the same time, the non-invasive approach is also particularly helpful in patients with chronic diseases. It allows non-invasive but very targeted follow-up of affected lung regions and, thanks to the visually impressive color-coded maps, comprehensive surveillance and a quick understanding of the course of the disease.

## 4 Conclusion

Functional lung imaging is a powerful approach for the targeted monitoring of pulmonary diseases. The use of MRI without exposure to ionizing radiation is particularly advantageous in pediatrics, with various options available for functional imaging. The functional NCE-MRI techniques are among the most innovative techniques with ongoing development. Their easy applicability on standard clinical MRI scanners without the need of a specialized set-up and specific breathing maneuvers or breath-holds makes them highly attractive for widespread use.

However, in order to allow comparison between different centers, the inconsistencies between different examination protocols and approaches to compute and report outcome values (even within the same techniques) should be addressed. Although VDP and QDP have been established as main outcome parameters, their calculation still varies substantially. Accordingly, there is an urgent need for expert consensus on technical standards required to be met when using NCE-MRI, underpinned by primary superiority studies where appropriate. Further, future objectives that need to be addressed are availability on devices of different manufacturers and field strengths, applicability even in neonates or infants and the use in clinical multi-site trials with large study samples.

Regarding the practical use of functional NCE-MRI, the studies presented illustrate its enormous potential for clinical decision-making. Assessing visual and numerical information on local pulmonary function allows a targeted examination and follow-up of affected lung regions as well as a better understanding of pathophysiology in complex diseases. Indications for therapeutic interventions or treatment adjustments can be determined accordingly.
