# Supplementary material for: Non-contrast enhanced functional lung MRI in children: systematic review
Source: Front Pediatr. 2025 Jul 22;13:1568172. doi: 10.3389/fped.2025.1568172 (PMC12322896; doi:10.3389/fped.2025.1568172)
Supplement: Supplementary file 1 [file Datasheet1.docx]

Online Supplement Material

**Non-contrast enhanced functional lung MRI in children: systematic review**

**I Review based on systematic literature research**

*Search methods and selection criteria*

We used the following information sources for our literature research without applying any language restrictions: MEDLINE® ALL (Ovid), Embase (Ovid), Cochrane Library (Wiley), ClnicalTrials.gov, International Clinical Trials Registry Platform ICTRP. For all databases, the data of last request was 12^th^ of August 2024. We aimed to find publications on MP-MRI, PREFUL-MRI, SENCEFUL-MRI or FD-MRI for functional imaging of the lung in children and used appropriate search strategies. Of 96 reports assessed for eligibility, we excluded 76 reports due to duplicates, thematic or age mismatch or because the findings were records on registered trials, conference contributions or review articles highlighting the functional MRI techniques of interest only in small subsections. In order not to overlook any publications in this innovative research area with evolving names and key phrases, all included articles were also screened for relevant references cited to add to the set (n=10).

*Detailed search strategy*

To identify potentially relevant publications on the topic, a search strategy was designed, and investigated in MEDLINE, Embase, Cochrane Library, ClinicalTrials.gov and ICTRP. The core concepts of the search strategy consisted of 1. Aspects regarding lungs 2. MP-, PREFUL-, SENCEFUL and FD-MRI, and 3. children. A medical information specialist developed an initial search strategy in Medline and tested it against a list of core references to ensure key publications were included. After refinement, the information specialist set up the search strategy for each information source based on database-specific index terms and free text. The free text search included synonyms, acronyms, and similar terms. No database-provided limits have been applied in any sources considering study types, languages or any other formal criteria. The search was first run on 26/04/2023 and last updated on 13/008/2024. The results were deduplicated using the automated deduplication tool deduklick (https://www.risklick.ch/products/deduklick/)^^[[1]](#footnote-1)^^. Results of the first searches were subtracted from the update search by following the Bramer method.^^[[2]](#footnote-2)^^

| **Search date** | **Database searched** | **Platform** | **Years of coverage** | **Records** |
| --- | --- | --- | --- | --- |
| 15 March 2024 | Medline | Ovid | 1946 - 2024 March 14 | 25 |
| 15 March 2024 | Embase | Ovid | 1974 - 2024 March 14 | 74 |
| 15 March 2024 | Cochrane Database of Systematic Reviews | Wiley | 1992 - present | 0 |
| 15 March 2024 | Cochrane Central Register of Controlled Trials | Wiley | 1992 - present | 0 |
| 15 March 2024 | ClinicalTrials.gov | NLM | 1997 - present | 10 |
| 15 March 2024 | ICTRP | WHO | 2007 - Present | 3 |
|  |  |  | Sum of references | 112 |
|  |  |  | Removed duplicates | -10 |
|  |  |  | **Total references** | **102** |

**Searches per database**

**Ovid MEDLINE(R) ALL <1946 to August 12, 2024>**

Search date: 131 August 2024

1 (lung* or pulmon* or bronchi* or alveol* or pneumo* or respirat*).ti,ab,kw. 2133310

2 exp Respiratory Tract Diseases/ 1805940

3 1 or 2 2966984

4 (senceful* or preful* or matrix-pencil* or MP-mri* or fourier decomposit*).mp. 887

5 exp adolescent/ or exp child/ or exp infant/ or (infant disease* or childhood disease*).ti,ab,kf. or (adolescen* or babies or baby or boy? or boyfriend or boyhood or child* or girl? or infant* or juvenil* or kid? or minors or minors* or neonat* or neonat* or newborn* or new-born* or paediatric* or peadiatric* or pediatric* or perinat* or preschool* or puber* or pubescen* or school* or teen* or toddler? or underage? or under-age? or youth*).ti,ab,kf,hw. 5283596

6 3 and 4 and 5 25

**Embase <1974 to 2024 August 12>**

Search date: 13 August 2024

1 (lung* or pulmon* or bronchi* or alveol* or pneumo* or respirat*).ti,ab,kw. 2843306

2 exp respiratory tract disease/ 3184400

3 or/1-2 4301659

4 (senceful* or preful* or matrix-pencil* or MP-mri* or fourier decomposit*).mp. 1708

5 exp *adolescence/ or exp *adolescent/ or exp *child/ or exp *childhood disease/ or exp *infant disease/ or (adolescen* or babies or baby or boy? or boyfriend or boyhood or child or child* or child*3 or children* or girl? or infant* or juvenil* or juvenile* or kid? or minors or minors* or neonat* or newborn* or new-born* or paediatric* or peadiatric* or pediatric* or perinat* or preschool* or puber* or pubescen* or school or school child* or school* or schoolchild* or schoolchild* or teen* or toddler? or underage? or under-age? or youth*).ti,kf. 3350121

6 3 and 4 and 5 74

**Cochrane Database of Systematic Reviews and Central Register of Controlled Trials**

Search date: 13 August 2024

#1 (lung* OR pulmon* OR bronchi* OR alveol* OR pneumo* OR respirat*):ti,ab,kw 224748

#2 [mh "Respiratory Tract Diseases"] 92757

#3 #1 OR #2 252888

#4 (senceful* OR preful* OR matrix-pencil* OR MP-mri* OR (fourier NEXT decomposit*)):ti,ab,kw 346

#5 [mh adolescent] OR [mh child] OR [mh infant] OR infant*:ti,ab,kw OR child*:ti,ab,kw OR childhood:ti,ab,kw OR (infant NEXT disease*):ti,ab,kw OR (childhood NEXT disease*):ti,ab,kw OR adolescen*:ti,ab,kw OR babies:ti,ab,kw OR baby:ti,ab,kw OR boy:ti,ab,kw OR boys:ti,ab,kw OR boyfriend:ti,ab,kw OR boyhood:ti,ab,kw OR girlfriend:ti,ab,kw OR girlhood:ti,ab,kw OR child*:ti,ab,kw OR girl:ti,ab,kw OR girls:ti,ab,kw OR infan*:ti,ab,kw OR juvenil*:ti,ab,kw OR kid:ti,ab,kw OR kids:ti,ab,kw OR minors:ti,ab,kw OR neonat*:ti,ab,kw OR neo-nat*:ti,ab,kw OR newborn*:ti,ab,kw OR new-born*:ti,ab,kw OR paediatric*:ti,ab,kw OR peadiatric*:ti,ab,kw OR pediatric*:ti,ab,kw OR perinat*:ti,ab,kw OR preschool*:ti,ab,kw OR puber*:ti,ab,kw OR pubescen*:ti,ab,kw OR school*:ti,ab,kw OR teen*:ti,ab,kw OR toddler:ti,ab,kw OR toddlers:ti,ab,kw OR underage:ti,ab,kw OR underage:ti,ab,kw OR under-ages:ti,ab,kw OR youth*:ti,ab,kw 396576

#6 #3 AND #4 AND #5 0

**ClinicalTrials.gov**

Search date: 13 August 2024

Condition or Disease:

lung OR lungs OR pulmon OR pulmonary OR respiratory

Other terms:

fourier decomposition OR senseful OR senceful OR preful OR matrix-pencil OR mp-mri

Eligibility Criteria:

Child (birth-17)

Studies found: 10

**ICTRP (https://trialsearch.who.int/)**

Search date: 13 August 2024

Search Strategy:

(lung* OR pulmon* OR bronchi* OR alveol OR pneumo* OR respirator*) AND (fourier decomposition OR senseful OR senceful OR preful OR matrix-pencil OR mp-mri)

Search results: 3

**Supplemental Table 1 Detailed overview on over published reports on functional NCE-MRI of the lung applied in the pediatric setting.**

In some cases, the division between main and secondary results was not made in the publications themselves, but was included in this table for better readability.

| **Disease group** | **technique** | **scanner** | **n coronal slices** | **assessed regional function** | **classification** | **main outcome parameter** | **additional outcome parameters** | **number included in analysis** | **age** | **main study aim (to assess…)** | **main results** | **secondary results** | **author** | **year** | **institute** |
| --- | --- | --- | --- | --- | --- | --- | --- | --- | --- | --- | --- | --- | --- | --- | --- |
| **Cystic Fibrosis** | FD [later: MP] | 1.5T | whole lung covered | perfusion | 0.8 Median | ● defect percentage (DP) based on relative perfusion | 3-point-functional score, assessed per lung field either automated or visual. Tresholds for score set by defect percentage (0: <7,5% or no defects resp.1: <50%and >=7,5% or <50% resp., 2: >=50%) | 34 | 4.08 [3,75] (median, IQR) | validation of FD MRI [later: MP-MRI] against DCE in young patients with CF | • equivalent diagnostic information to DCE ● validation given by mean difference, upper and lower limits of intra-reader-agreements between functional score ● highest agreement found between FD and DCE MR data processed by using automated scoring ●automated scoring more precise than visual scoring, especially at DP of around 50% | • high concordance between visual and automated scoring allowing for percentage-based analysis ● defect percentage (DP) based on fractional ventilation | Bauman | 2013 | Heidelberg |
|  | MP | 1.5T | whole lung covered | · ventilation · perfusion | 0.8 Median | ● defect percentage (DP) based on fractional ventilation and relative perfusion |  | · CF: 40 · HC: 12 | · CF: 12.0 (6-18) · HC: 12.0 (5-17) (median, range) | ● to assess whether indices from functional MRI differ between healthy controls and patients with CF ● analysed the correlation between this new functional MRI method and N2-MBW in children with CF ● | · VDP and QDP sign. higher in CF compared to HC · strong correlation of VDP with LCI, FEV1 z-score, FCV z-score and RV/TLC · correlation of QDP with LCI and FEV1 z-score · correlation of morphology score (and subscorse) with LCI and FEV1 · strong inter-reader correlation for morphology score in patients with CF | · minimal structural changes in morph. MRI in 4 HC, wide range in patients with CF · LCI sign. higher in CF, to a lesser degree also difference in spiroemetry and bodyplethysmography outcomes · | Nyilas | 2017 | Bern |
|  | MP | 1.5T | whole lung covered | • ventilation • perfusion | 0.8 Median | ● defect percentage (DP) based on fractional ventilation and relative perfusion |  | • CF: 23 • HC: 12 | • CF: 13.5 +- 3.7 • HC: 11.9 +- 4.1 | ● 24h reproduciblity of VDP and QDP in healthy and children with CF ● relate reproducibility of VDP and QDP to reproducibility of LCI ● confirm feasability and correlation between MRI and lung function | • (very) good agreement between repeated measurements in CF and HC • short term changes of VDP or QDP > +-4.4% should be considered greater than biological variability | • in CF: VDP, QDP and LCI elevated compared to HC • in CF: correlation of VDP with FEV1, LCI (not found in healthy controls), also correlation of Eichinger score and LCI ● feasible in all study pariticpants | Nyilas | 2019 | Bern |
|  | MP | 1.5T | whole lung covered | • ventilation • perfusion | 0.8 Median | ● defect percentage (DP) based on fractional ventilation and relative perfusion |  | • CF: 25 • HC: 10 | ● CF: 13.5 (+-3.6) ● HC: 11.1 (+-4.0) mean (SD) | repeatability and reproducibiltiy of lung segmentation (by human observers and an artifical neural network (ANN)) and impact on outcome parameters | ● high intraobserver repeatability of human observers (no/unrelevant bias of outcomes) ● perfect intraobserver repeatability of ANN ● good interobserver reproducibility of human observers (small but significant bias of DP --> avoidable by using ANN) ● reproducbility of ANN and human observer as good as between two different human observers (also small but significant bias of DP) ● validation of ANN | ● strong correlation of VDP and QDP with LCI for all segmentation approaches ● higher intra-reader variation of regression coefficients (DP to LCI) for QDP than VDP ● no relevant difference of R2 for regression DP to LCI between the different segmentation approaches ● AIC and AIC difference best for ANN (for VDP and QDP) | Willers | 2021 | Bern |
|  | MP | 1.5T | whole lung covered | • ventilation • perfusion | 0.8 Median | ● defect percentage (DP) based on fractional ventilation and relative perfusion | ● defect distribution index (DDI) | ● CF: 53 | 11.9 [6.1 to 17.8] median [interquartile range] | quantifiy homogeneity of defect distribution using a novel measure | • strong correlation between computed defect distribution index (DDI) and visual scoring system for defect distribution | • moderate to strong correlation of DDI_V and DDI_Q with DP, FEV1, LCI, Eichinger score (exception: DDI_V and FEV1 --> DDI_V more sensitive/complementary to FEV1) | Valk | 2021 | Bern |
|  | MP | 1.5T | whole lung covered | • ventilation  • perfusion | 0.8 Median | ● defect percentage (DP) based on fractional ventilation and relative perfusion |  | 30 | 12.5+- 2.3 (mean, sd) | ● short-term functional changes to nebulized inhalation with salbutamol in children with CF ● identify clinical factors that best predict a positive effect of salbutamol on an individual level | • on a populational level: FEV1 and QDP improved, LCI improved marginally, VDP stable • on an individual level: some patients improved | • no clinical factors associated with more pronounced response found | Kieninger | 2022 | Bern |
|  | MP | 1.5T | whole lung covered | • ventilation  • perfusion | 0.8 Median | ● defect percentage (DP) based on fractional ventilation and relative perfusion | ● defect distribution index (DDI) | 24 | 13.8, [8.6 to 17.2] | ●the ability of functional and structural MRI to monitor treatment response of ELX/TEZ/IVA in children with CF ● to compare treatment response of ELX/TEZ/IVA to previous baseline evolution of lung function and MRI ● to assess possible associations between changes in MRI and lung function | • VDP, QDP, DDI_V, DDI_Q sign. improved • Eichinger score sign. improved • FEV1 and LCI sign. improved • Also improvements on individual level compared to previous baseline evolution (MP MRI might in some patients be more sensitive) | • sing. correlation of deltaVDP with deltaFEV1 and deltaEichinger, delta DDI_Q with deltaLCI | Streibel | 2023 | Bern |
|  | MP | 1.5T | whole lung covered | · ventilation  · perfusion | 0.8 Median | ● defect percentage (DP) based on fractional ventilation and relative perfusion |  | · CF: 39 | 21.6 +- 10.7 (mean, SD) 8 - 45 (range) | To compare MP-MRI with established morpho-functional MRI and spirometry in patients with CF. | · MP MRI perfusion score and QDP correlated strongly with contrasted-enhanced MRI perfusion score · MP MRI ventilation score and VPD showed strong inverse correlation with FEV1pp | · MP MRI perfusion score correlated strongly with QDP · MP MRI ventilation score correlated strongly with VDP · MP MRI perfusion score and MP MRI ventilation score correlated strongly · QDP and VDP correlated strongly | Doellinger | 2024 | Berlin |
|  | FD (later: PREFUL) | 1.5T | 3 | ventilation | no DP |  | Fractional ventilation (median, quartile coefficient of dispersion) | ● CF. 16 ● HC: 12 | ● CF: 14.5 [12.8 to 16.8] ● HC: 25.5 [22.3 to 35.3] (median, interquartile range) | ● feasibility of functional lung MRI (DCE, oxygen enhanced, FD) to detect and quantify regional parenchymal differences beteween adolescents with CF and HC ● feasibility of functional lung MRI to detect changes in regional lung function two hours after a single hypertonic saline treatment in comparison to spirometry and MBW | ● ability to discriminate (AUC of ROC-curve 0.89) ● fractional ventilation sign. decreased and more heterogeneous in upper lobes and whole lung in CF compared to HC ● median T1-values at room air and at oxygen sign. lower and more heterogenouse in upper lobes and whole lung in CF compared to HC ● no significant treatment effect two hours after singel treatment with hypertonic saline (neither fuctional MRI parameters nor MBW) | ● correlation between functional MRI assessemtents of regional ventilation (FD, T1 oxygen enhanced) and perfusion (DCE) ● no correlation bewteen pulmonary function tests and functional lung MRI parameters and Eichinger score | Kaireit | 2017 | Hannover |
|  | 2D PREFUL | 1.5T | not mentioned | ● ventilation ● perfusion ● VQ match | ● FV: Median-4*SD ● Q: Median-0.5*SD | ● defect percentage (DP) based on fractional ventilation and relative perfusion | ● DP based on time to peak (TTP) of perfusion and respiratory cycle (segmentation of DP by 50+- 10% deviation for V-TTP and >95th percentile of the volunteer data (>200ms) for Q-TTP) ● regional flow volume loops (RFVL) | ● CF:1 ● HC:2  ● other disease: 2 | • CF:16 • HC: 22, 25 ● other disease: 42, 44 | feasability of PREFUL | • feasible to assess regional ventilation and perfusion • visual agreement of perfusion maps with DCE MRI ● possible to quantify regional ventilation dynamics using V-TTP and RVFL analysis • possible to obtain V/Q (TTP) maps | • in patient with CF: heterogeneous ventilation and many regions with abnormal perfusion | Voskrebenzev | 2018 | Hannover |
|  | 2D PREFUL | 1,5 T | 4 | ● ventilation | 90th percentile*0.4; binning (ventilation defect region and ventilation defect region + low ventilation region) | ● defect percentage (DP) based on fractional ventilation | ● DP based on regional flow volume loops (RFVL) (segmentation of DP in RFVL cross correlation (CC) maps by 90th percentile and by linear binning) ● quartile coefficient of dispersion (QCD) as a measure of ventilation heterogeneity (calculated for RV and RFVL) ● VDP of combined RV and RVFL-CC maps | CF: 8; HC: 6; COPD: 20 | ● CF 15 (13-17) ● HC 24 (22-30) ● COPD 69 (60-72) (md, 25, 75 pc) | ● correlation of PREFUL with 129Xe and lung function tests ● comparison of PREFUL derived ventilation parameters and tresholding techniques | ● good spatial agreement between PREFUL and 129Xe ● VDP (of all types), RFVL_CC and ventilation heterogeneity increased in patients compared to healthy controls ● correlation between PREFUL-, 129Xe- and spirometry outcomes ● combined (RV and RFVL) defect map showed better regional agreement and correlation with 129Xenon defct maps than each PREFUL defect map alone | ● combined (RV and RFVL) VDP sign. higher compared to 129Xe VDP ● no syst difference between RV VDP_90*4 and RV VDP_LB ● sign. difference between RFVL-CC VDP_90*4 and RFVL-CC VDP_LB ● sign. higher RV and visually higher flow rates of reference voxels in patients compared to those in healthy volunteers ● RFVL of healthy volunteers have round shape, altered shape in patients (fluctuations of flow rate and concave shape of expiratory limb) ● sign. difference between combined VDP_90*4 and combined VDP_LB ● sign. difference between 129Xe VDP_90*4 and 129Xe VDP_LB | Kaireit | 2021 | Hannover |
|  | 2D PREFUL | 3T | 1 | ventilation | k-means | ● defect percentage (DP) based on fractional ventilation | mean slice fractional ventilation | • CF(stable): 6 • CF(PEx):11 • HC: 10 | • CF(stable): 11.7 +- 1.8 • CF(PEx): 15.2 +- 1.9 • HC: 11.5 +- 2.5 (mean, sd) | feasability of PREFUL in pediatric CF participans with a range of disease severity, including those undergoing a pulmonal exacerbation (PEx) | • feasible for ventilation • concordance with 129Xe MRI results (even though different absolute DP values, but correlation and good dice coefficients in all groups) • sign. correlation of DP PREFUL and DP 129Xe with LCI and FEV1 | ● in CF pEx: VDP of PREFUL and of Xenon sign. elevated compared to HC and mean FV of PREFUL decreased compared to HC (these findings not in CF stable) ● sign. difference of LCI between heathy and CF (stable and PEx) ● CF stable had normal FEV1, CF Pex had sign. reduced FEV1 compared to HC and CF stable | Couch | 2021 | Toronto |
|  | 2D PREFUL | 3T | ● 1 ● several for Xe | ventilation | k-means | ● defect percentage (DP) based on fractional ventilation |  | 8 | 15 [11 to 16] (median, 95% CI) | explore whether PREFUL MRI can be used to track response to i.v. antibiotics for a pulmonary exacerbation | ● improvement of VDP (PREFUL and Xe) following treatment of Pex | ● FEV1, PREFUL VDP and Xe VDP improved after treatment (LCI did not) ● PREFUL VDP correlated with Xe VDP, FEV1, LCI ● no sign. correlation between relative improvement of PREFUL VDP and Xe VDP ● no correlation between changes in PREFUL VDP and FEV1 and LCI | Munidasa | 2021 | Toronto |
|  | 2D PREFUL | 1.5T | • whole lung • and focus on 3 central slices separately | perfusion | median normalized, 2% | ● defect percentage (DP) based on relative perfusion |  | CF center 1: 10 CF center 2: 6 | ● center 1: 12-18 ● center 2: 19-47 (ranger | compare perfusion-weighted PREFUL MRI across two different sites and two differenct scanners in patients with CF with a range of age and lung disease severity | • high concordance with DCE (sing. correlation and median spatial overlap of 79.4%) • similar results observed in both centers | • sign. correlation of PREFUL QDP and FEV1 (underpowered for DCE QDP) ● few patients with QDP differences >10% between PREFUL and DCE ● sign. difference of median spatial overlap between centres for whole lung, not for three central slices | Behrendt | 2022 | Hannover, Sheffield |
|  | 2D PREFUL | 1.5T | 7 | ventilation | binning (ventilation defect region and ventilation defect region + low ventilation region) | ● defect percentage (DP) based on fractional ventilation |  | ● CF center 1: 24 ● CF center 2: 7 ● HC center 2: 6 | ● CF center 1: 23.3 +- 10.2 ● CF center 2: 15.4 +- 2.0 ● HC center 2: 25.8 +- 4.4 (mean, sd) | relationship between 129Xe and PREFUL ventilation in patients with a broad spectrum of CF using data of 2 centres | ● sign. correlations between PREFUL VDP and Xe VDP and centres ● sign. correlations of FEV1 z-score and LCI with Xe VDP, Xe VDP + LVP, PREFUL VDP, PREFUL VDP + LVP ● Xe VDP had sign. stronger correlation with LCI than PREFUL VDP, linear regression relationship of FEV1 and Xe VDP vs. PREFUL VDP not sign. different | ● pooled over 2 centres, compared to Xe, ● PREFUL VDP tended to overestimate VDP for milder disease and over-/underestimate for severe disease (not apperant in VDP + LVP) ● more false positive in PREFUL VDP, Xe more sensitive for early-stage lung disease ● regional similarities and differences of Xe and PREFUL maps, but throughout the lung generally siilar | Marshall | 2023 | Hannover, Sheffield |
|  | 2D PREFUL | 3T | ● 1 ● several for Xe | ventilation | • k-means | ● defect percentage (DP) based on fractional ventilation and cross-correlation | ● regional flow volume loops (RFVL) ● regional flow volume loop cross correlation (RFVL_CC) ● VDP from both: regional fractional ventilation and RFVL_CC (treshold: <90%) and combined | • CF: 15 • HC:7 | • CF: 15 [13 to 16]  (md, 25,75pc) • HC: 15 [12 to 15] (md, 25,75pc) | ● intra- and interscan repeatability of PREFUL in children with stable CF and healthy participants ● comparison of PREFUL results to Xe MRI , FEV1 and LCI | • very good intravisit repeatability for all VDP paramters of PREFUL and Xe-MRI • good to moderate intervisit repeatability ● intervisit repeatability of VDP < intravisit repeatbility  ● VDP from Xe with lower intervisit variablity than from PREFUL | • in CF: PREFUL, Xe and lung function outcomes impaired compared to HC ● in CF: stronger corellation of Xe VDP to FEV1 and LCI than PREFUL VDP  ● in CF: sign. correlation of intervisit percentage change of Xe-MRI, PREFUL MRI and LCI, not of FEV1 • correlation DP PREFUL and DP 129Xe • correlation DP PREFUL (several types) and DP 129Xe with FEV1 and LCI, but stronger for 129Xe • FEV1 and LCI also very good intervisist repeatability --> PREFUL might be more sensitive ● VDP_CC with strongest correlation and regional agreement to VDP_Xe and LCI and highest reatability | Munidasa | 2023 | Toronto |
|  | · 2D PREFUL  · 3D PREFUL | 1.5T | ● 2D: n.a. ● 3D: k | ventilation | ● VDP_rvent: 90th percentile*0.4 ● VDP_cc: 0.9 CC | ● defect percentage (DP) based on fractional ventilation and cross-correlation | ● fractional ventilation ● regional flow volume loop (RFVL) ● regional flow volume loop cross correlation (RFVL_CC) | 23 | 21.0 ± 9.3 (mean, SD) | to investigate if the ventilation parameters derived by 3D PREFUL are suitable to  measure response to ETI therapy and their association with  improvements in clinical outcome measures in CF patients. | ● all PREFUL ventilation markers sign. improved after ETI ● MRI global, morphology and perfusion socres improved after ETI ● RVent did not significantly improved | ● FEV1, MEF25 and LCI improved after ETI ● no sign. correlation between abs. and rel. changes in PREFUL MRI vent. Parameters with FEV1, MEF25, LCI and MRI scores ● sing. Correlation between relative change of SD RVent with relative change of LCI ● further correlations between PREFUL outcome parameters and lung function test outcomes ● post treatment VDP corellated sign. with spirometry, LCI, global score, morph. score and perfusion score ● no sign. correlation of VDP_CC with global and morphology score ● PREFUL CC values sing. correlated with FEV1, MEF25 and LCI | Klimes | 2024 | Hannover |
|  | 2D PREFUL | 1.5T | ● whole lung covered | ● ventilation ● perfusion | ● VDP_rvent: 90th percentile*0.3 ● VDP_cc: 0.9 CC ● QDP: 90th percentile*0.3 | ● ventilation: defect percentage (DP) based on fractional ventilation and cross-correlation ● perfusion: defect percentag (DP) based on estimating the voxelwise proton density and calculating the median signal decay toward the steady state as a reference for the perfusion amplitude |  | 23 (subsample of previous multi-center study with n=91) | 18 [14-24.5] (median, IQR) | to investigate if PREFUL MRI–derived quantitative ventilation and perfusion measures can assess changes in people with CF after initiation of ETI therapy and how these measures relate to established clinical and MRI-derived parameters | ● VDP_RV, QDP, Eichinger score, and LCI significantly improved after ETI treatment | ● Median Perfusion and dynamic ventilation heterogeneity improved after ETI ● Area of no VDP no QDP increased after ETI ● mild to moderate correlations between change in FVL_CM (PREFUL) and change in LCI, ppFEV1, MEF25 ● no correlation between change in PREFUL perfusion paramters and changes in lung function parameters or Eichinger global or subscores, including perfusion score ● only MEF25 correlated with the subscore of bronchial wall thickening | Dohna | 2024 | Hannover |
|  | SENCEFUL | 1.5T |  | ventilation | n.a., no DP |  | quantitative ventilation | • CF: 20 • HC: 20 |  | clinical feasability of SENCEFUL for quantitative ventilation imaging | • feasible • in CF: quantitative ventilation lower than in HC, especially in upper lung parts | • in CF: diseminated small ventilation deficits as most frequent pattern • in CF: correlation QV with FVC, RV, FEV1 | Veldhoen | 2017 | Würzburg |
|  | SENCEFUL | 1.5T | whole lung covered | perfusion |  |  | ● qualitative assessment by radiologists: 5-point Likert scale per quadrant rating perfusion deficits or aberrations of the pulmonary perfusion phase ● quantitative assessment: peak-to-Offset ratio of perfusion phase values (maximum value divided by the mean of the outer 10% values) per subject, slice and quadrant basis | • CF: 20 • HC: 20 | • CF: 23.6 +- 11.2  • HC: 24 +- 10.5  (mean, SD) | feasability of SENCEFUL for detection of pulmonary perfusion deficits in patients with proven CF | • feasible • in CF: higher perfusion deficit score compared to HC (qualitative assesement) • in CF: lower phase peak-to-offset ratio compared to HC (quantitative assesment) | • good ICC for perfusion deficit score • similar qualitative results in perfusion weighted maps and perfusion phase maps • correlation of peak-to-offset ratio with FEV1, FEF75, FEV1/FVC, MMEF | Kunz | 2021 | Würzburg |
|  | FD | 1.5T | 2 | • ventilation • perfusion | n.a., no DP |  | ● semi quantitative score of defet extents on V and Q maps (0 = abscence/negligible, 1= <50%, 2= >50%) regrading right/left lung, superior, medion or inferior regions ● mean and coefficient of variation (CV) of signal intensitiy (SI) of ventilation and perfusion | 12 | 14 [6.5 to 32] (median, range) | evaluate the response to treament of ABPA in CF patients based on FD-MRI functional changes | ● extent ventilation and perfusion defects sign. decreased after ABPAtreatment (semiquantitative defect score) ● mean SI of V sign. increased after ABPA treament, no sign. changes of QSI_mean, VSI_cv and QSI_cv | ● morphological Bhalla score sing. increased (improved) after treatment ● before treatment no sign. correlation between PFT parameters and functional or structural lung MRI outcomes ● after treatment sign. correlation of ventilation defect extents and PFT obstructive parameteres (inverse), QSI_mean and FEV1%, QSI_cv and FEF 25-75% (inverse), Bhalla score and PFT obstructive parameters ● very good ICC for functional and structural visual scores and quantitative outcomes ● overlap between regions with structural deficits and V or Q defects (especially bronchiectasis with mucus plugging)  ● pre- and post-ABPA treament changes in VSI_cv adn QSI_cv sign. correlated with changes in FEV1% ● no correlation Bhalla and visual/quantitative functional V and Q outcomes | Benala | 2024 | Munic |
| **Bronchopulmonary dysplasia and Fetal Growth Restriction** | MP | 1.5 T | whole lung covered | ● ventilation ● perfusion | 0.8 (0.7?) Median | ● defect percentage (DP) based on fractional ventilation and relative perfusion |  | ● without FGR: 20 ● with FGR: 20 | ● without FGR: 18.4 (6.6) ● with FGR: 18.4 (6.6) (mean; SD) | to examine the association between FGR and lung development independent of other important risk factors by comprehensive assessment of lung function and structure in monochorionic twin pairs with selective FGR | ● low birth weight associated with lower FEV1 and FVC, but FEV1/FVC similar in both groups ● low birth weigh associated with lower TLC and higher RV/TLC ● no group differences of LCI and FENO ● minimal structural abnormalities in morphological MRI, no group differences ● no group differences in VDP and QDP | ● no difference in FRC-MBW and S-cond between groups ● elevated S-acin associated with lower birth weight group ● no association of VDP or QDP with FEV1, FVC or S-acin | Salem | 2022 | Bern |
|  | 2D PREFUL | 1.5 T | 1 central 2 additional (anterior and posterior) | ventilation | 90th percentile*0.4 | ● defect percentage (DP) based on fractional ventilation | ● mean ventilation (%) ● regional flow volume loops (treshold: 90%) | ● preterm_BPD: 6 ● preterm_noBPD: 6 | 40.0 weeks (md) post-menstrual age, [38.0 to 49.0] (IQR) | feasibility to quantifiy lung ventilation in perterms at neonatal age with and without BPD | ● VDP sign. higher in preterm_BPD compared to preterm_noBPD ● feasible, watch out for rotational motion | ● no sedation, but "feed and swaddle" ● flow-volume loop correlation metric also assesed ● might be: specific regions with ventilation defects with increasing severity of BPD, different homogeneity of regional ventilation maps with increased severity of BPD | Dyke | 2023 | New York, |
|  | FD (analyzed using an in-house developed algorithm) | 1.5T | whloe lung covered | • ventilation • perfusion | n.a. |  |  | • preterm_BPD: 11 • preterm_noBPD: 9  • HC: 9 | • preterm_BPD: 11.0 (7.2 to 15),  • preterm_noBPD: 11.1 (10.7 to 12.6) • HC: 11.6 (8.8 to 12.8) (median, range) | ● develop a chest MRI protocol on a standard 1.5 T clinical scanner to assess lung structure and function in children with and without BPD at school age ● validate MRI findings against spirometry | • structural MRI: in BPD bronchopathy and hyperintensity and total diseased lung elevated compared to HC and noBPD • structural MRI: in noBPD bronchopathy most common finding, % of all abnormalities elevated compared to HC | • all structural MRI images (different sequences used) had diagnostic quality, UTE, T2 weighted PROPELLER, and ZTE sequences performed best • correlation of %diseased lung (and AAD) and FEV1, FEF75 and FEV1/FVC • in BPD: lower FEV1, FEF75 and FEV1/FVC than HC compared to noBPD • FD MRI (exploratory): ventilation and perfusion defects corresponded to hypointense regions on expiratory MRI, but only large hypointense regions detected (not minor e.g. mosaic perfusion), plus some moving artefacts due to slice thickness of 15mm, image qualitiy in some children not good enough) • FD MRI (exploratory): ventilation and perfusion defects found in some children | Elders | 2022 | Rotterdam |
| **Other pulmonary diseases** | MP | 1.5T | whole lung covered | ● ventilation ● perfusion | 0.8 (0.7?) Median | ● defect percentage (DP) based on fractional ventilation and relative perfusion |  | 30 | 13.4 (10.4 - 17.1) (md, IQR) | ● prevalence and extent of lung function abnormalities in patients with PCD ● concordance between lung function and structural outcomes in patients with PCD | ● all participants with structural lung abnormalities (main ones: bronchiectas/ bronchial wall thickening, mucus plugging) ● elevated VDP in 52% and QDP in 78% of participants, abnormal FEV1 in 27%, abnormal LCI in83% ● concordance of bronchiectasis, mucus plugging and elevated LCI ● no sign. correlation between LCI and VDP or QDP |  | Nyilas | 2018 | Bern |
|  | • 2D PREFUL  • 3D PREFUL | 3T | • 1 (2D) • k (3D) | • ventilation • perfusion • match | k-means | ● defect percentage (DP) based on fractional ventilation and relative perfusion | ● pulmonary blood flow based on 3D scan ● regional ventilation ● regional flow volume loops (RFVL) ● regional flow volume loop cross correlation (RFVL_CC) | 10 | >=36 weeks, =< 24months | investigaet feasability and quality of structural and functional 3T MRI in neonates and infants with no clinically defined cardiorespiratory disease | ●feasible (structural, 2D PREFUL and 3D PREFUL) ● no dataset considered nondiagnostic, good overall diagnostic quality | • high quality of structrual scans, good repeatability of visual score • in 2 patients: structural abnormalities, elevated VDP, slightly elevated QDP • 78% of vessels detectable in pulmonary blood flow imaging | Zanette | 2022 | Toronto |
|  | 2D PREFUL | 0.55T | 1 | ● ventilation ● perfusion ● match | ● perfusion: 2% ● ventilation: 40% of 90th percentile ●flow-volume loop: 0.9 | ● defect percentage (DP) based on fractional ventilation, relative perfusion and regional flow volume loops | ● mean perfusion and ventilation ● regional flow volume loops (RFVL) ● regional flow volume loop cross correlation (RFVL_CC) ● V/Q defect, V/Q match | ● long Covid: 25 ● recovered: 29 ● HC: 9 | ● long Covid: 12 +- 3 ● recovered: 11 +- 3 ● HC: 10 +- 3 | to characterize morphologic and functional changes of lung parenchyma in children and adolescents after SARS-CoV-2 in low-field-strength MRI compared to healthy controls | ● only one patient in post-Covid showed any morphological changes (linear atelectasis) | ● post-Covid: VDP and V/Q sign. higher compared to HC ● long Covid and recovered: VDP and V/Q sign. higher compared to HC ● increase in VDP, QDP and V&Q according to time from infection ● | Heiss | 2023 | Erlangen |
| **Localized lung alterations** | MP | 1.5T | whole lung covered | ● ventilation ● perfusion | 0.8 (0.7?) Median | ● defect percentage (DP) based on fractional ventilation and relative perfusion |  | 5 | 7.32 (5.25 to 10.3) (mean, range) | functionality of expanded lung tissue in children with congenital pulmonary airway malformation using lung function tests and functional lung MRI | ● impairment of ventilation and perfusion in the specific areas of expanded lung tissue ● not covered by standard pulmonary function tests |  | Willers | 2021 | Bern |
|  | MP | 1.5T | whole lung covered | • ventilation • perfusion • match | 0.8 (0.7?) Median | ● defect percentage (DP) based on fractional ventilation and relative perfusion | ● defect distribution index (DDI) | • large CDH: 6 • small CDH: 7 • HC: 13 | • large CDH: 10.9 +- 1.4 • small CDH: 9.9 +- 3.0 • HC: 10.5 +- 2.5 | long-term pulmonary outcome of children with CDH overall and side-specific | • in large CDH: impaired overall lung function compared to HC and small CDH • in large CDH: pronounced impairment of the affected lung side | • in large CDH: decrease in FEV1 due to reduced vital capacity upon hyperinflation rather than an obstruction • in CDH: sign. corr. FEV1 and RV/TLC with VDP_aff, QDP_aff, VQD_match_aff | Streibel | 2023 | Bern |
|  | SENCEFUL | 3T | 6 | • ventilation • perfusion | n.a. | no DP | fractional ventilation and regional perfusion | ● hypoplasia of left lung artery: 1 ● HC: 22 | ● patient: 17 ● HC: 20-30 (range) | ● feasability of SENCEFUL | ● SENCEFUL advantageous to only difference of inspiration vs. exspiration and FD (better functional contrast, less artifacts) ● good accordance with DCE (but outperformed) and FD results ● in patient: left lung with reduced perfusion but not reduced ventilation | • influence of matrix size: increasing the spatial resolution lowers the SNR/functional contrast of the maps • multiple averaging of phase enconding steps (25x) results in good functional maps (high SNR, high contrast, low artifact level), | Fischer | 2014 | Würzburg |

1. Borissov N, Haas Q, Minder B, et al. Reducing systematic review burden using Deduklick: a novel, automated, reliable, and explainable deduplication algorithm to foster medical research. Syst Rev. 2022;11(1):172. Published 2022 Aug 17. doi:10.1186/s13643-022-02045-9 [↑](#footnote-ref-1)
2. Bramer W, Bain P. Updating search strategies for systematic reviews using EndNote. J Med Libr Assoc. 2017;105(3):285-289. doi:10.5195/jmla.2017.183 [Add to Citavi project by DOI] [↑](#footnote-ref-2)
